# Supplementary material for: Effectiveness and Safety of Ayurvedic Medicines in Type 2 Diabetes Mellitus Management: A Systematic Review and Meta-Analysis
Source: Front Pharmacol. 2022 Jun 8;13:821810. doi: 10.3389/fphar.2022.821810 (PMC9213670; doi:10.3389/fphar.2022.821810)
Supplement: Supplementary file 1 [file DataSheet1.zip › Supplementary Material/Forest and Funnel Plots/Zingiber officinale/Body weight.pdf]

Zingiber officinale - Body weight

Study	Intervention N	Intervention Mean	Intervention SD	Comparator N	Comparator Mean	Comparator SD	Mean Diff. with 95% CI	Weight (%)
Mahluji 2013	28	79.10	11.40	30	76.90	14.10	2.20 [-4.43, 8.83]	9.30
Arablou 2014a, Arablou 2014b	33	66.10	8.20	30	66.00	7.70	0.10 [-3.84, 4.04]	26.34
Azimi 2014, Azimi 2016	41	79.21	6.15	39	78.48	6.87	0.73 [-2.12, 3.58]	50.15
Shidfar 2015	22	80.02	13.20	23	78.20	13.40	1.82 [-5.96, 9.60]	6.76
Arzati 2017, Zarezadeh 2018	23	77.93	11.19	22	76.68	14.04	1.25 [-6.15, 8.65]	7.46
**Overall**							0.81 [-1.21, 2.83]	

Heterogeneity: $\tau^2 = 0.00$, $I^2 = 0.00\%$, $H^2 = 1.00$

Test of $\theta_i = \theta_j$: Q(4) = 0.38, p = 0.98

Test of $\theta = 0$: z = 0.79, p = 0.43

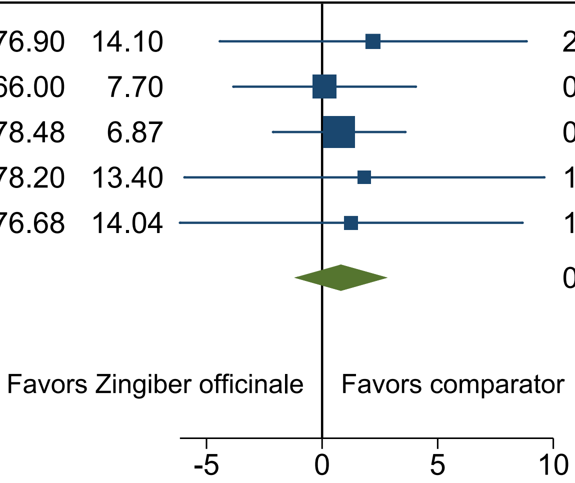

Random-effects REML model
